# Comparison of lobectomy and sublobar resection for stage I non-small cell lung cancer: a meta-analysis based on randomized controlled trials

**DOI:** 10.3389/fonc.2023.1261263

**Published:** 2023-10-04

**Authors:** Genlin Lu, Zhiyi Xiang, Yan Zhou, Senjie Dai, Fei Tong, Renya Jiang, Min Dai, Qiufeng Zhang, Difeng Zhang

**Affiliations:** ^1^ General Surgery Department, Longyou County People’s Hospital, Quzhou, Zhejiang, China; ^2^ The First Clinical Medical College, Zhejiang Chinese Medical University, Hangzhou, Zhejiang, China; ^3^ Anesthesia Surgery Center, The First Affiliated Hospital of Ningbo University, Ningbo, Zhejiang, China; ^4^ The Second Clinical Medical College, Zhejiang Chinese Medical University, Hangzhou, Zhejiang, China; ^5^ Hepatobiliary Surgery Department, Quzhou City People’s Hospital, Quzhou, Zhejiang, China; ^6^ Department of Orthopaedics, Ningbo Yinzhou No. 2 Hospital, Ningbo, Zhejiang, China

**Keywords:** stage I, non-small cell lung cancer, lobectomy, sublobar resection, meta-analysis, overall survival, disease-free survival

## Abstract

**Background:**

This meta-analysis aimed to compare the prognostic between lobectomy and sublobar resection in patients with stage I non-small cell lung cancer (NSCLC).

**Methods:**

We conducted a detailed search in PubMed, Embase, Web of Science, and the Cochrane Library for randomized controlled trials (RCTs) comparing the prognosis of lobectomy and sublobar resection for stage I NSCLC, with the primary outcomes being overall survival (OS) and disease-free survival (DFS).

**Results:**

A total of 2222 patients were included in the 5 RCTs. The results showed no statistical difference in OS (HR=0.87, p=0.445) and DFS (HR=0.99, p=0.918) between patients who underwent lobectomy and sublobar resection during the total follow-up period. In terms of dichotomous variables, there were no statistical differences in OS (relative ratio [RR]=1.05, p=0.848) and DFS (RR=1.21, p=0.075) between the two groups during the total follow-up period, as well as 5-year OS (RR=0.96, p=0.409) and 5-year DFS (RR=0.95, p=0.270). In addition, subgroup analysis showed a better prognosis for non-adenocarcinoma patients with sublobar resection than lobectomy (HR=0.53, p=0.037), but also an increased cause of cancer death (not limited to lung cancer) (RR=1.56, p=0.004).

**Conclusion:**

Our results showed that for stage I NSCLC, lobectomy is usually not a justified operation.

**Systematic review registration:**

https://www.crd.york.ac.uk/prospero/display_record.php?ID=CRD42023407301, identifier CRD42023407301.

## 1 Introduction

As the second most widespread cancer and the leading cause of cancer deaths in the world, lung cancer has a cancer diagnosis rate of approximately 11.4% and a cancer mortality rate of 18.0% (1). Because of the advent of computed tomography (CT), more non-small cell lung cancers (NSCLC) are being diagnosed at an early stage (2). Lobectomy has long been the standard surgical treatment for stage I NSCLC (3), and patients who undergo lobectomy have an ideal overall survival (OS), with patients achieving a 5-year OS and 10-year OS of 77% and 70%, respectively, in one study (4). In theory, sublobar resection may offer anatomical and functional advantages over lobectomy because it preserves more lung tissue and improves the quality of patient survival, so there are proposals to reduce the extent of resection and preserve more lung function. However, another concern about sublobar resection is whether the prognosis of patients will be affected, and more studies are needed to compare the difference in prognosis between the two.

Liu et al. published a meta-analysis in 2014 comparing OS between lobectomy and sublobar resection in stage IA NSCLC, including 12 studies from 1993 to 2013 and found that OS was not as robust with sublobar resection as with lobectomy (5). In 2021, Lv et al. did another meta-analysis, including 12 studies from the establishment of the database to 2019. The analysis showed that patients with stage I NSCLC undergoing sublobar resection demonstrated poorer OS, while disease-free survival (DFS) was similar for both approaches (6), but neither article was based on randomized controlled trials (RCTs). Recently, the results of a new high-quality RCT study were published which showed similar prognostic outcomes for sublobar resection and lobectomy (7). Given the above situation, we believe that there is a compelling need to re-evaluate sublobar resection and lobectomy. Therefore, we performed a meta-analysis based on published RCTs to compare the differences between lobectomy and sublobar resection in prognosis in patients with NSCLC.

## 2 Methods

This study was conducted according to the Preferred Reporting Items for Systematic Reviews and Meta-Analyses (PRISMA) 2020 statement: an updated guideline for reporting systematic reviews (8), registered in the “International Prospective Register of Systematic Reviews” (PROSPERO) in 2023 (CRD42023407301). The objective was to evaluate the prognosis of lobectomy and sublobar resection for stage I NSCLC by RCTs.

### 2.1 Literature search strategy

From the time of database establishment to March 2023, two researchers conducted a systematic and exhaustive screening of PubMed, Embase, Web of Science, and the Cochrane Library databases for articles on lobectomy and sublobar resection for NSCLC, using the following keywords:((lobectomy OR lobar resection) AND ((sublobar resection OR limited resection) OR (wedge AND segmentectomy)) AND ((lung cancer OR pulmonary cancer OR carcinoma of lung OR pulmonary carcinoma OR lung carcinoma OR lung neoplasms OR lung adenocarcinoma OR cancer of lung)). In particular, references to relevant literature were manually searched to avoid omitting any potentially relevant studies.

### 2.2 Inclusion and exclusion criteria

According to the PICOS principles, the criteria for inclusion in the studies were as follows: 1) patients were diagnosed with clinical stage I NSCLC (tumor size equal to or less than 3 cm, no regional lymph node metastasis), sublobar resection was extended to lobectomy if N1 disease is found during surgery; 2) intervention and control were sublobar resection and lobectomy; 3) outcomes of relevant included but were not limited to OS, DFS, recurrence rate, etc.; and 4) the included studies belong to the RCTs.

The exclusion criteria for this study were: 1) the full text of the study was not available; 2) the study data were not available, including the protocol; 3) when updating published articles for the same study cohort, studies that included the most recent or largest population were selected.

### 2.3 Data extraction

Data extraction was performed independently by 2 researchers according to a pre-designed form. For eligible studies, the following relevant information was extracted: 1) study characteristics: author, year of publication, country, sample size, and registration number; 2) participant characteristics: including tumor stage, histological typing, age, gender, follow-up time, etc.; and 3) survival outcomes applied for comparison.

### 2.4 Quality assessment

Two researchers used the Cochrane Collaboration’s tools to assess the quality of RCTs. Three indicators of “high risk”, “low risk”, and “unclear risk” were used to assess random sequence generation, allocation concealment, blinding of participants and personnel, blinding of outcome assessment, incomplete outcome data, selective reporting, and other sources of bias. Two researchers, after discussion, will discuss and resolve differences in the evaluation, and bring in a third person when necessary.

### 2.5 Statistical analysis

This meta-analysis was performed using Review Manager, v.5.3, and Stata software, v.12.0. Hazard ratio (HR) and 95% confidence interval (CI) were used to evaluate continuous variables, and the relative risk (RR) and 95% CI were used to evaluate dichotomous information. Heterogeneity was calculated with the I^2^ statistic; I^2^>75% was considered severe heterogeneity, >50% and <75% high heterogeneity, >25% and <50% moderate heterogeneity, and <25% low heterogeneity. Due to the diversity of the population included in this study, a random-effects model was used uniformly to combine the results with the premise of improving the credibility of the results. A p-value <0.05 in a two-sided test is statistically significant (9). When more than ten studies were included, publication bias was investigated using Begg’s test (10), and sensitivity analysis was conducted to evaluate the stability of the results.

## 3 Result

### 3.1 Description of the studies

**Figure 1:**
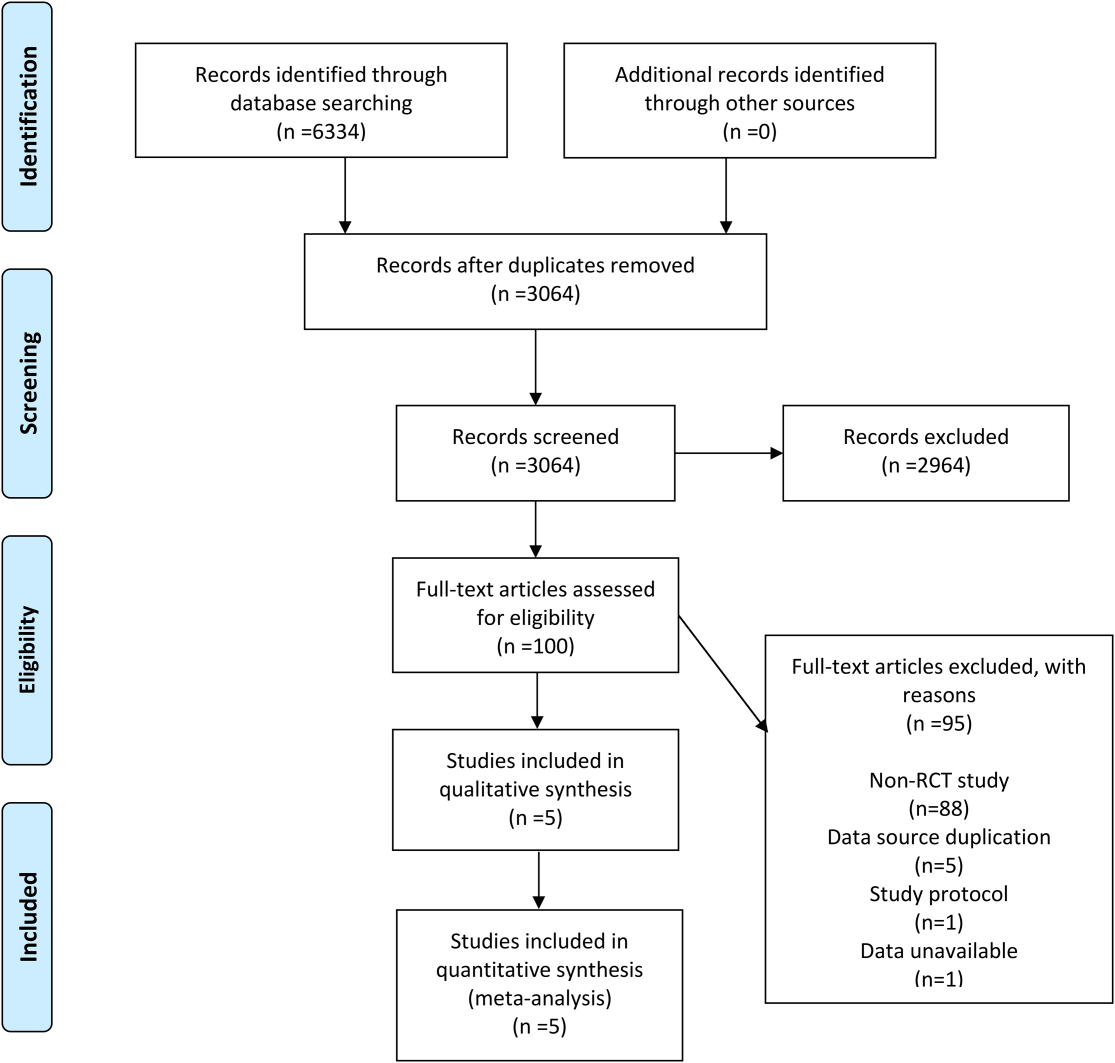
Flow diagram of selection.

6334 records were retrieved across the four databases using the set search strategy and no additional records were retrieved from other sources. After removing duplicates, 3064 records remained, and 2964 irrelevant articles were excluded by reviewing the titles and abstracts of the articles. After browsing the complete text, 95 articles were excluded, of which 88 were not RCTs, 5 due to duplication of data sources, 1 for being a research protocol, and 1 owing to unavailable data. In the finals, 5 RCTs (7, 11–14) were included in our meta-analysis. In **Figure 1**, the flowchart demonstrates the detailed process and the exclusion criteria.

Between 1995 and 2023, 5 RCTs compared survival outcomes of patients with stageINSCLC after lobectomy and sublobar resection. Of all patients, 1100 underwent sublobar resection, and the other 1122 underwent lobectomy. In three studies, sublobar resection included both segmental and wedge resection; the remainder included only segmental resection. In addition, all but one of the studies were for stage IA NSCLC with follow-up beyond 5 years and all provided OS and DFS. The characteristics of the studies included in this meta-analysis are outlined in **Table 1** and **Supplementary Table 1**.

**Table 1:** Characteristics of all the studies included in the meta-analysis.

Author	Year	Country	Treatment regimens		Number of patients		Neoplasm staging	Follow-up (years)	Outcomes
			Experiment	Control	Experiment	Control			
Robert J. Ginsberg	1995	America	Segmentectomy +wedge resection	Lobectomy	122	125	I	>4.5	DFS, OS
Terumoto Koike	2016	Japan	Segmentectomy +wedge resection	Lobectomy	33	32	IA	>5	DFS, OS
Nasser K Altorki	2022	Australia, Canada, America	Segmentectomy +wedge resection	Lobectomy	340	357	IA	>5	DFS, OS
Georgios Stamatis	2022	Germany, Switzerland, Austria	Segmentectomy	Lobectomy	53	54	IA	5	DFS, OS
Hisashi Saji	2022	Japan	Segmentectomy	Lobectomy	552	554	IA	>5	DFS, OS

I, tumor size equal or less than 3 cm; IA, tumor size smaller than 2 cm in longest dimension; DFS, Disease-free survival; OS, overall survival.

### 3.2 Risk of bias in the included studies

The quality assessment of the included studies is presented in **Supplementary Figure 1** and **Supplementary Figure 2**. The quality of each RCT was evaluated using the Cochrane Collaboration’s tool. All studies were assessed as low risk in terms of blindings of outcome assessment and incomplete outcome data. Most studies were assessed as low risk in three aspects: random sequence generation, allocation concealment, and selective reporting. A small number were considered an unclear risk. However, in terms of blinding of participants and personnel, three studies were of unclear risk, and the remaining two were of high risk, which was determined by the nature of the intervention. For other biases, the included studies were assessed as unclear risks.

### 3.3 Prognostic analysis

**Figure 2:**
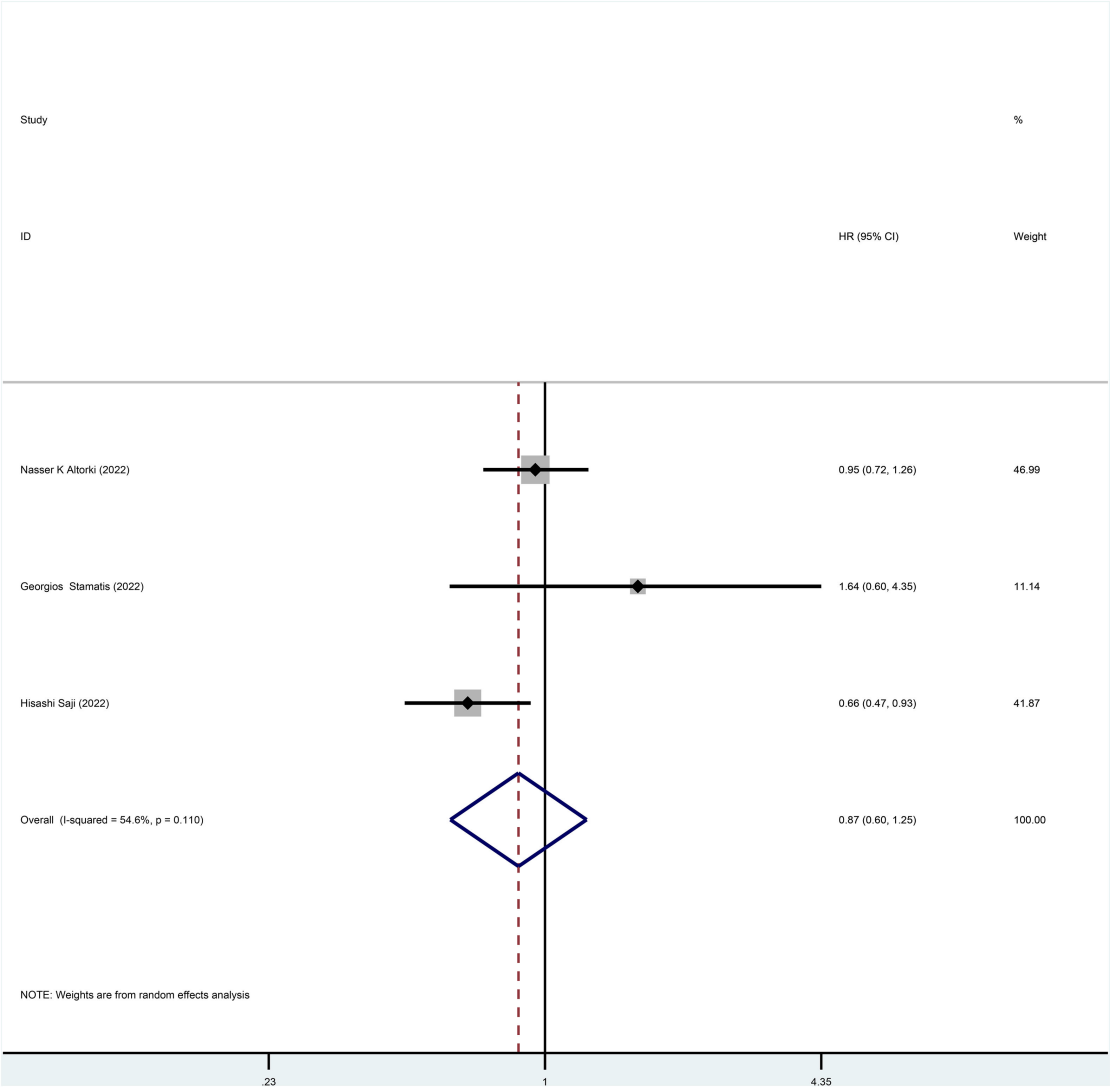
Forest plot of meta-analysis of the effects of sublobar resection and lobectomy on overall survival in stage I NSCLC (HR perspective, p=0.445).

**Figure 3:**
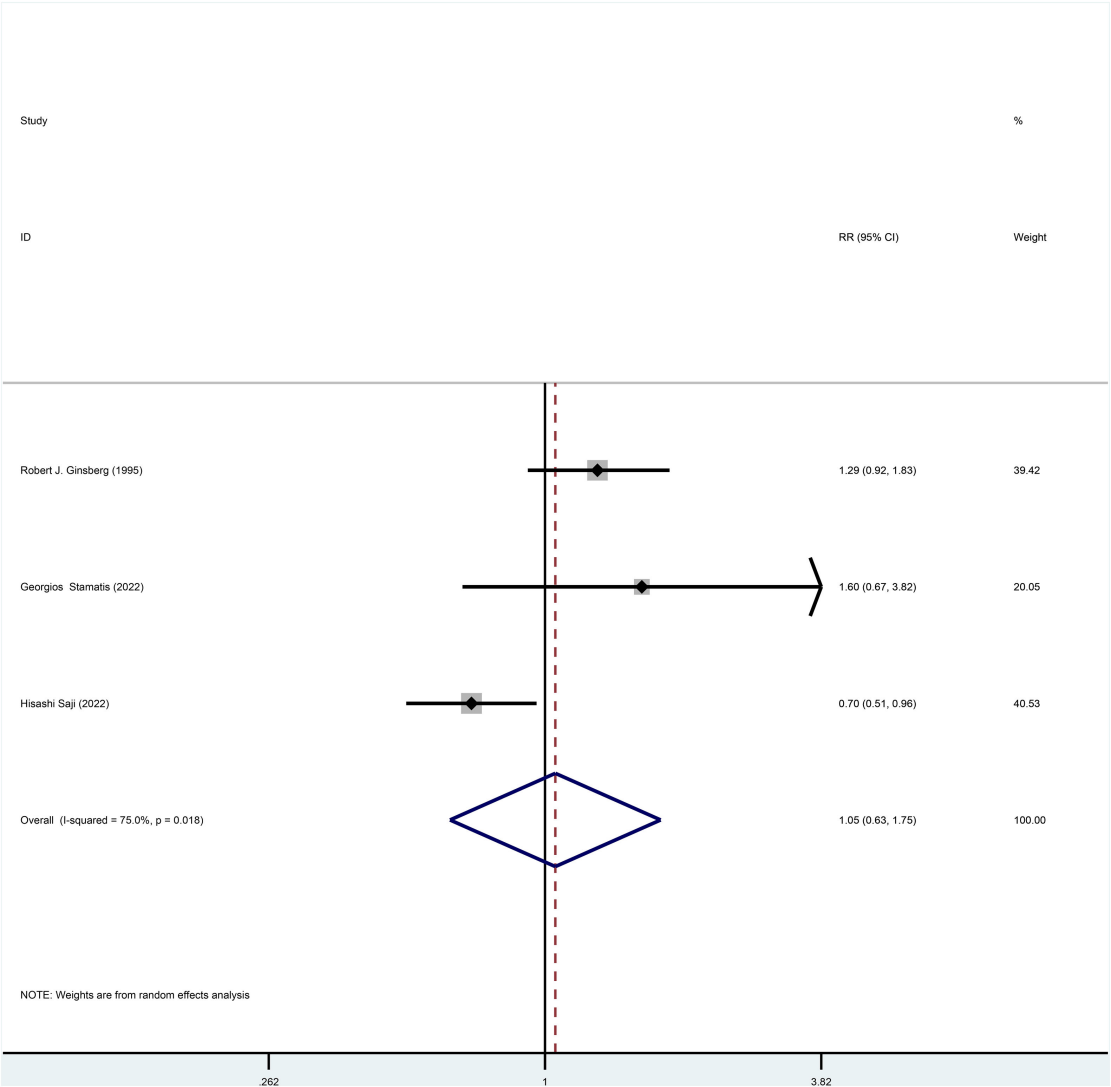
Forest plot of meta-analysis of the effects of sublobar resection and lobectomy on overall survival in stage I NSCLC (dichotomous variable perspective, p=0.848).

Three studies reported HR for OS in patients with stage I NSCLC who underwent sublobar resection versus lobectomy throughout the follow-up period, with pooled results indicating no difference in OS (HR=0.87, 95%CI=0.60-1.25, p=0.445) (**Figure 2**). In addition, from the perspective of dichotomous variables, the results showed no significant difference between the two groups in terms of OS during the follow-up period (RR=1.05, 95%CI=0.63-1.75, p=0.848) (**Figure 3**), but by a higher heterogeneity (I ^2^ = 75%, p=0.018). Five studies offered 5-year OS, and the results showed no difference in 5-year OS between the two groups (RR=0.96, 95%CI=0.89-1.05, p=0.409) (**Supplementary Figure 3**); the results were also highly heterogeneous (I ^2^ = 70%, p=0.010).

**Figure 4:**
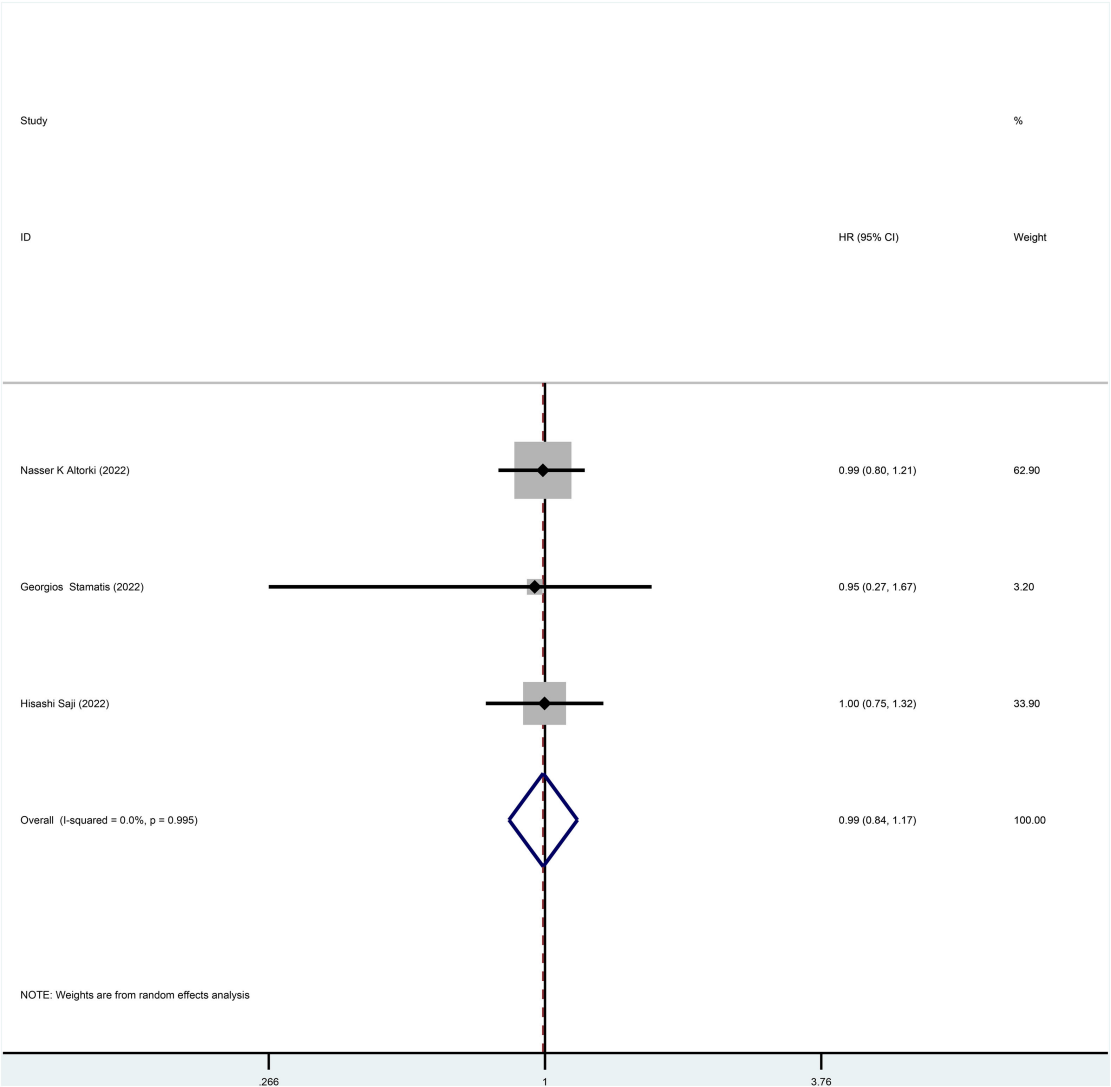
Forest plot of a meta-analysis of the effects of sublobar resection and lobectomy on disease-free survival in stage I NSCLC (HR perspective, p=0.918).

**Figure 5:**
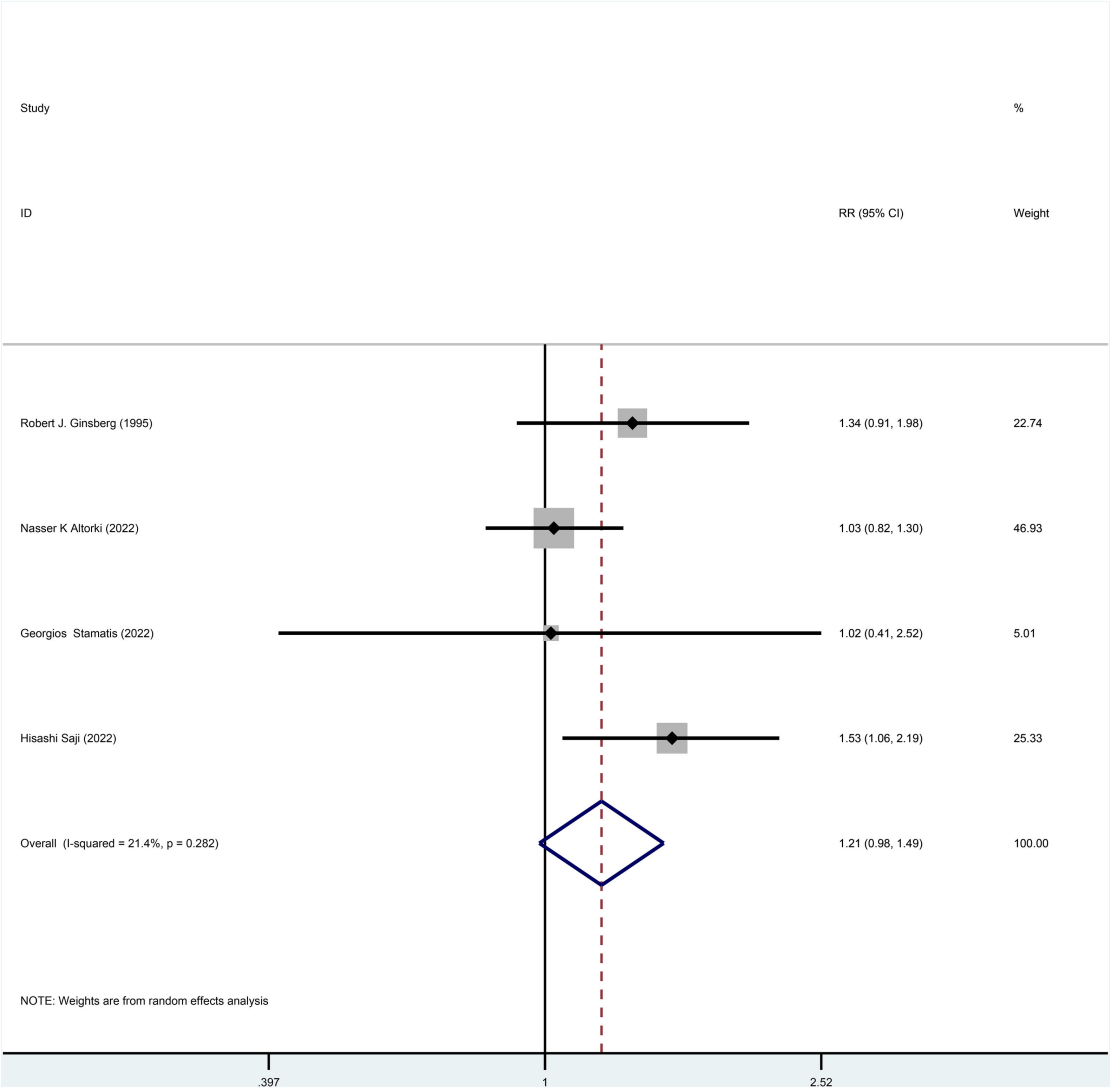
Forest plot of a meta-analysis of the effects of sublobar resection and lobectomy on disease-free survival in stage I NSCLC (dichotomous variable perspective, p=0.075).

Three studies reported HR for DFS in patients with stage I NSCLC throughout the follow-up period, with pooled results showing no statistical difference in DFS between patients who underwent sublobar resection and those who underwent lobectomy (HR=0.99, 95%CI=0.84-1.17, p=0.918) (**Figure 4**). No heterogeneity was detected in the studies included (I^2^ = 0). Moreover, from the perspective of dichotomous variables, there was also no difference in DFS among the two groups at the overall follow-up (RR=1.21, 95%CI=0.98-1.49, p=0.075) (**Figure 5**). Five studies delivered 5-year DFS and the statistical outcomes showed no significant differences in the 5-year DFS between the two groups (RR=0.95, 95%CI=0.86-1.04, p=0.270) (**Supplementary Figure 4**).

### 3.4 Subgroup analysis

Predefined subgroup analyses were performed, as detailed in **Table 2**. Subgroup analyses of OS considering gender, histological typing, and cause of death, were conducted. Subgroup analysis regarding gender showed no significant differences in OS between patients undergoing sublobar resection and lobectomy in the male (HR=0.99, p=0.981) or female groups (HR=1.45, p=0.534). For patients with adenocarcinoma, no difference was found in the OS after surgery between the two groups (HR=1.2, p=0.673). However, it is worth noting that for patients with non-adenocarcinoma, OS was statistically better for those who underwent sublobar resection than lobectomy (HR=0.53, p=0.037). When cancer cause of death (not limited to any cancer) was analyzed as the primary outcome, patients who underwent sublobar resection had a lower OS than those who underwent lobectomy (RR=1.56, p=0.004). For other causes of death (non-cancer), no difference in OS was observed between those who underwent sublobar resection and lobectomy (RR=1.13, p=0.552).

**Table 2:** Subgroup analysis of overall survival.

	No. of studies	HR/RR	95%CI	P	Heterogeneity	
					I^2^	P
Female	2	1.45	0.45, 4.61	0.534	82.8%	0.016
Male	2	0.99	0.37, 2.61	0.981	84.4%	0.011
Adenocarcinoma	2	1.26	0.43, 3.71	0.673	88.8%	0.003
Non-adenocarcinoma	2	0.53	0.30, 0.96	0.037	0	0.696
Death due to cancer	3	1.56*	1.15, 2.10	0.004	0	0.792
Death to other cause	3	1.13*	0.76, 1.69	0.552	0	0.959

HR, hazard ratio; RR, risk ratio; CI, confidence interval.

*The pooled effect size is RR.

## 4 Discussion

Our results showed no difference in prognosis between patients with stage I NSCLC who underwent lobectomy and sublobar resection, using OS and DFS as the primary endpoints. Previously, the results of Nakamura et al. showed that the two surgical approaches were comparable in terms of OS (15), which is consistent with the results of the present study, whereas the results of Lv et al. showed comparable results between the two only in terms of DFS (6), while lobectomy was superior to sublobar resection in terms of OS, which is inconsistent with the findings of the present study. The present study is the first meta-analysis based on published RCTs and the results have a high level of confidence.

Since the publication of the results of the LSCG trial in 1995 (11), lobectomy has become the standard procedure for early-stage lung cancer. The extent of resection for early-stage NSCLC remains a controversial issue, but in all surgical resections, whether lobectomy or sublobar resection, the principles of oncologic treatment should be strictly adhered to, including radical resection of the tumor, reducing surgical risk and preserving the patient’s organism as much as possible (16).

Our study showed no difference between the lobectomy and sublobar resection in OS and DFS over the total follow-up period in terms of the HR and the dichotomous variable perspective; 5-year OS and 5-year DFS were also comparable in terms of the dichotomous variable perspective. This may be due to the better prognosis of patients with stage I NSCLC, with data showing that the 5-year survival rate of patients with stageINSCLC in the United States was about 70% between 2001 and 2017 (17). Besides, a prospective trial of stage I NSCLC demonstrated a local regional recurrence rate of 2% and a 5-year survival rate of 91% in patients when the surgical margin distance was greater than the tumor size (18). Other studies have also demonstrated that the local recurrence rate after segmental resection for stage I NSCLC is in the range of 2%-8% (19–24). The key to sublobar resection is to ensure adequate margins, which are an important factor in local recurrence and prognosis. In the article by Georgios Stamatis et al., sublobar resection is an anatomical segmentectomy using a standardized protocol for anatomical segmentectomies. The segmentectomy by Hisashi Saji et al. includes one segmental resection and bi-segmental resection (including left tri-segmentectomy), excluding basal segmentectomy. The groups that performed sublobar resections in the remaining articles all performed segmental resections or wedge resections at the surgeon’s discretion. All sublobar resection groups in the included studies had negative margins confirmed by margin lavage cytology or frozen section examination. So it is speculated that sublobar resection is sufficient for the complete resection of the tumor and surrounding subclinical lesions in stage I NSCLC.

In addition, the low rate of lymph node metastasis in stage I NSCLC may also be another factor, with the results of related studies showing that the rate of lymph node metastasis in stage I NSCLC ranges from 3.2% to 14.5% (25–27). An RCT comparing lymph node sampling and complete lymph node dissection in the mediastinum showed no difference in postoperative survival and recurrence rates between these two approaches (28), and other studies have also shown that lymph node dissection performed in early-stage lung cancer has no effect on patient survival (29–31), and given these results, it can be hypothesized that performing sublobar resection resulting in less lymph node dissection may not affect prognosis.

Regarding the effect of gender on OS after two surgical approaches, the results of Kim et al. showed that gender was not a factor affecting the survival rate of both surgical modalities (32) and a propensity-matching analysis study by Zhou et al. showed that in women, the lobectomy group was superior to the sublobar resection group, while in men, there was no difference between the two surgical approaches (33). However, there was no difference in OS between male and female patients with stage I NSCLC who underwent lobectomy or sublobar resection in the subgroup of this study. Presumably, as the sample size included in the analysis increases, gender is no longer a factor affecting OS.

Concerning histological staging, our meta-analysis showed that for non-adenocarcinoma in stage I NSCLC, OS was better and statistically significant for sublobar resection than with lobectomy. In contrast, for adenocarcinoma, there was no difference in OS between lobectomy and sublobar resection. This may be attributed to the fact that adenocarcinoma is more often seen in women, and most of its occurrence is not due to tobacco, but more likely to the increased inhalation of oil-based cooking fumes, household pollutants, and industrial dust (34), and one study suggests that increased frequency of cooking fume inhalation may be an important factor in lung cancer in non-smoking women (35). These patients are young, their lung function is better and, in theory, the more lung tissue preserved by sublobar resection, the less it will contribute to the improvement of lung function. While squamous carcinoma predominates in the non-adenocarcinoma population, the main bronchial squamous cell carcinoma is in turn associated with male smokers (34), such an incidence population is associated with older age, poor cardiopulmonary function and a higher risk of serious comorbidities, while sublobar resection preserves more lung substance, theoretically preserving more postoperative lung function and potentially reducing short- and long-term pulmonary complications, thus improving patient’s OS.

Regarding the cause of death, the results of this study showed that the number of cancer deaths (not limited to lung cancer) was higher with sublobar resection than with lobectomy, with statistically significant results. Lung cancer probably accounts for the majority of the deaths. In addition to the possibility that cancer cells remaining at the surgical margin, it is also possible for lung cancer to spread through the air space (STAS). In 2015, the WHO defined “STAS” as the invasion of the airspace around the lung parenchyma by micropapillary, solid nests, or clusters of single cells beyond the tumor margin (18). Mino-Kenudson’s study indicated that the frequency of STAS can range from 15% to 56% in different cohorts as well as in tumor stages (36). Some studies reported that STAS is an important independent factor for recurrence after sublobar resection in early NSCLC (37–40). The mechanism may be that STAS in the alveolar space beyond the surgical margins goes undetected, leading to increased mortality from lung cancer. In addition, sublobar resection preserves more lung tissue than lobectomy, increasing the probability of secondary lung cancer in patients. Among non-cancer causes of death, sublobar resection could theoretically reduce the incidence of postoperative complications and reduce non-cancer mortality because of the preservation of lung function. However, the combined results of the two groups did not differ, and sublobar resection did not reduce the risk of non-cancer causes of death relative to lobectomy. This may be because comprehensive postoperative treatment reduced the non-cancer mortality in the lobectomy group and does not exclude the fact that the study’s included population had better lung function and that postoperative cardiopulmonary function was not severely affected even with lobectomy.

Besides, according to WHO statistics in 2019, cardiovascular disease has become the number one cause of death worldwide. Thus, considering competing mortality rates, survival rates for early-stage lung cancer are high, reaching 70% (17), while more patients die from heart disease, cerebrovascular disease, chronic obstructive pulmonary disease, and other non-tumor factors, resulting in a smaller percentage of deaths from cancer, which may explain why there is no difference in OS between lobectomy and sublobar resection, while sublobar resection has a higher cause of cancer death than lobectomy, but the non-cancer cause of death rate is comparable between the two.

There are some limitations to this study. A total of 5 RCTs to date were included to compare the prognosis of lobectomy and sublobar resection. The small number of articles makes them more susceptible to chance. More detailed subgroup analyses, such as the effect of race, age, and thoracoscopic surgery on OS, or the differences between the different types of sublobar resection and their indications are difficult to perform because of the limited nature of the data. Large samples of RCTs and more detailed data are still needed for more detailed subgroup analyses of groups, specific staging, and histology for specific surgeries, leading to more specific conclusions. Due to differences in the populations included in the study, there was some heterogeneity in some of the results.

In conclusion, this meta-analysis showed that for stage I NSCLC, lobectomy is usually not a justified operation. Gender was not a factor affecting OS for lobectomy and sublobar resection in stage I NSCLC, and sublobar resection in non-adenocarcinoma patients had a better OS, but at the same time, sublobar resection might increase the risk of cancer death (not limited to lung cancer).

## Data availability statement

The original contributions presented in the study are included in the article/**Supplementary Material**. Further inquiries can be directed to the corresponding author.

## Author contributions

GL: Writing – original draft, Conceptualization, Methodology. ZX: Writing – original draft, Methodology, Software. YZ: Data curation, Software, Writing – original draft. SD: Data curation, Methodology, Software, Writing – original draft. FT: Resources, Validation, Writing – original draft. RJ: Data curation, Methodology, Software, Writing – original draft. MD: Data curation, Validation, Writing – original draft. QZ: Resources, Validation, Writing – original draft. DZ: Conceptualization, Supervision, Validation, Writing – review & editing.

## Supplementary material

Supplementary Figure 1 Risk of bias summary of the included RCTs.

Supplementary Figure 2 Risk of bias graph of the included RCTs.

Supplementary Figure 3 Forest plot of meta-analysis of the effects of sublobar resection and lobectomy on 5-year overall survival in stage I NSCLC (dichotomous variable perspective, p=0.409).

Supplementary Figure 4 Forest plot of a meta-analysis of the effects of sublobar resection and lobectomy on 5-year disease-free survival in stage I NSCLC (dichotomous variable perspective, p=0.270).
