# Supplementary figures and images for: Comparison of lobectomy and sublobar resection for stage I non-small cell lung cancer: a meta-analysis based on randomized controlled trials
Source: Front Oncol. 2023 Oct 4;13:1261263. doi: 10.3389/fonc.2023.1261263 (PMC10582352; doi:10.3389/fonc.2023.1261263)

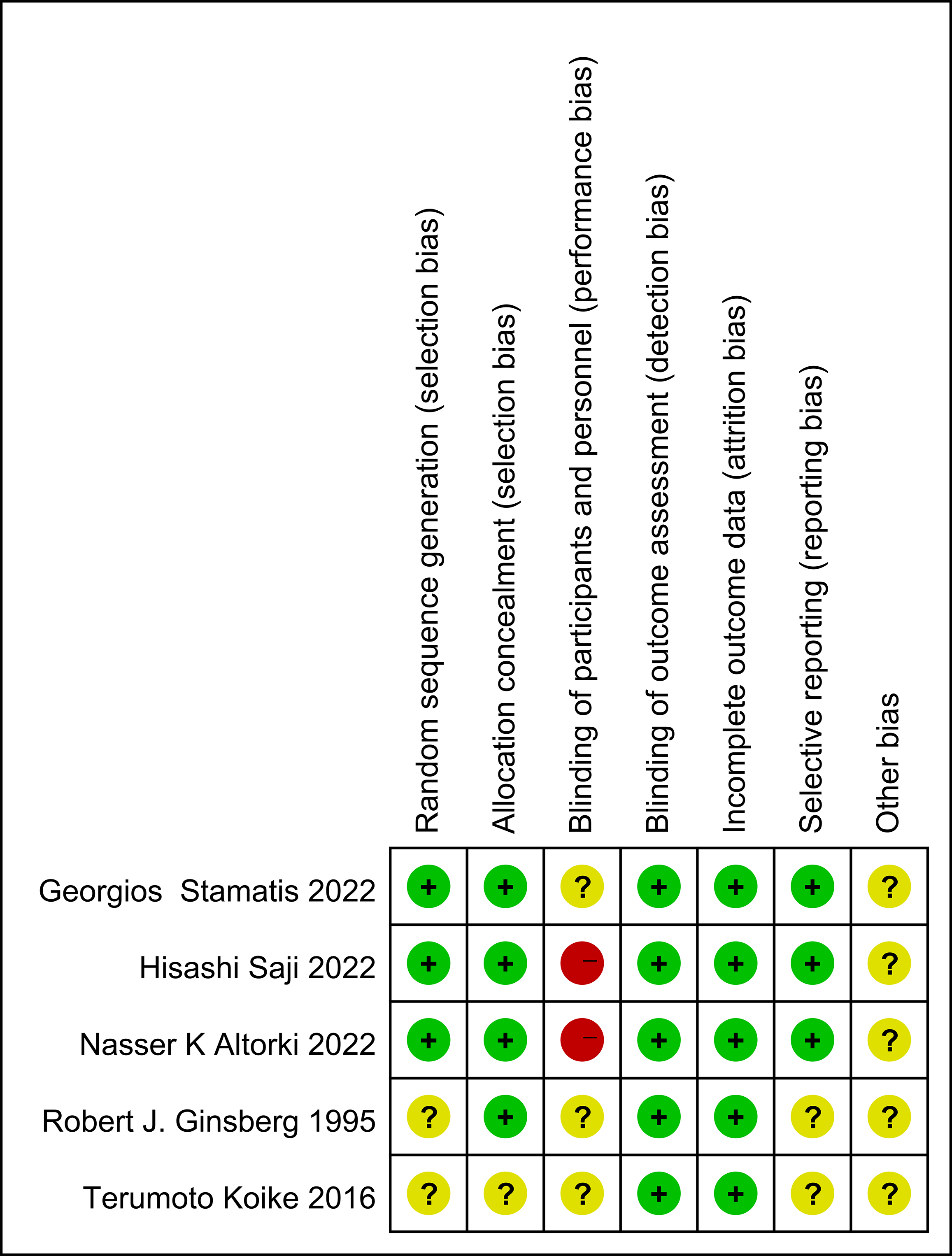

Supplement: Supplementary Figure 1 — Risk of bias summary of the included RCTs. [file Image_1.tif]

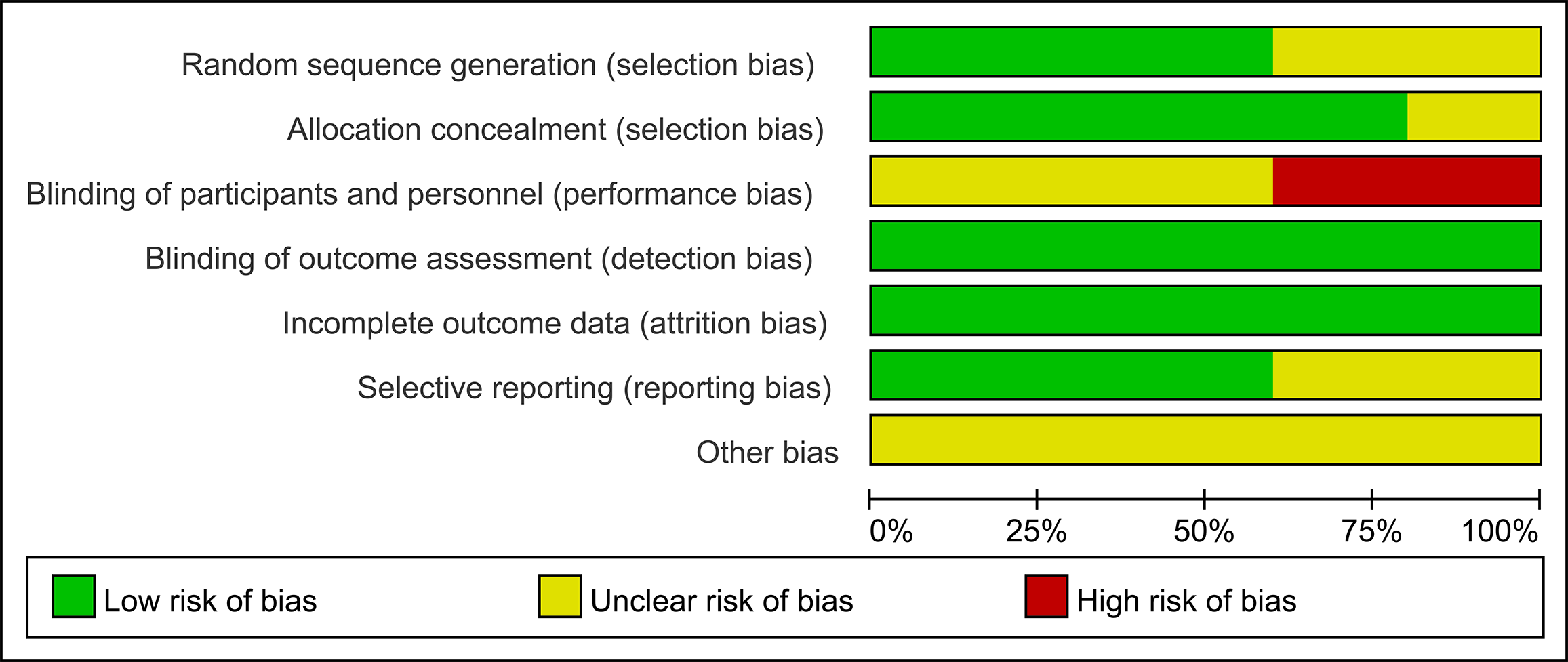

Supplement: Supplementary Figure 2 — Risk of bias graph of the included RCTs. [file Image_2.tif]

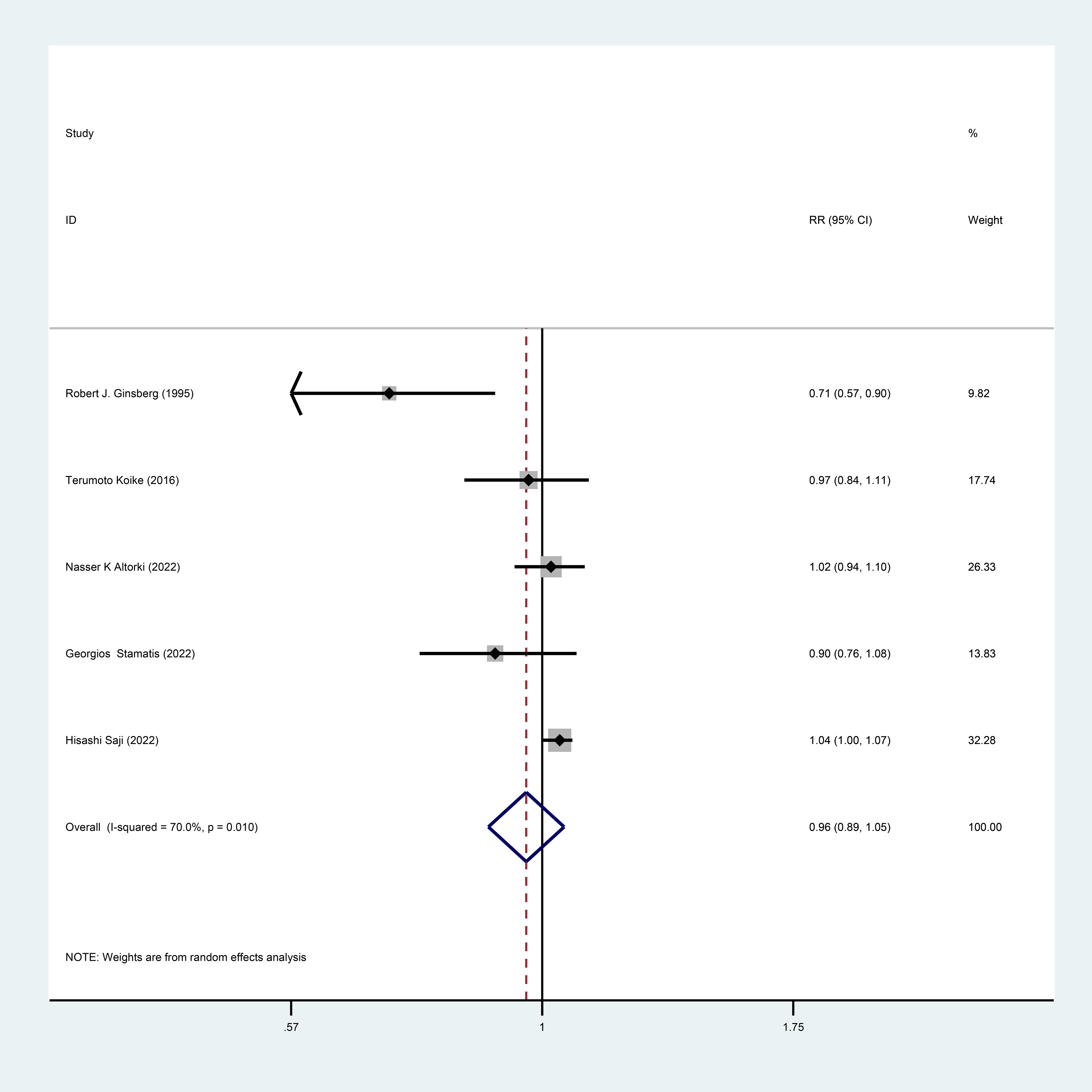

Supplement: Supplementary Figure 3 — Forest plot of meta-analysis of the effects of sublobar resection and lobectomy on 5-year overall survival in stage I NSCLC (dichotomous variable perspective, p=0.409). [file Image_3.tif]

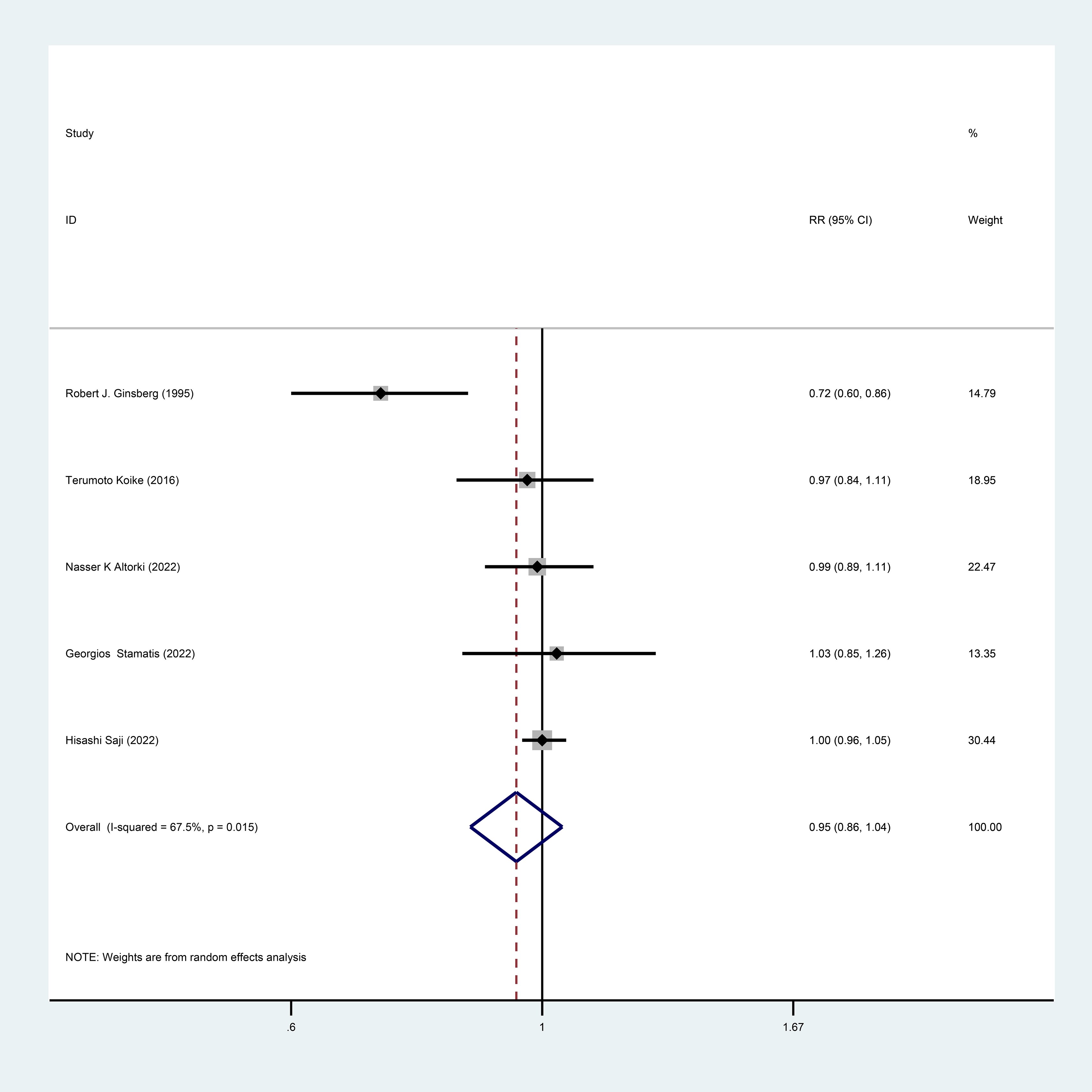

Supplement: Supplementary Figure 4 — Forest plot of a meta-analysis of the effects of sublobar resection and lobectomy on 5-year disease-free survival in stage I NSCLC (dichotomous variable perspective, p=0.270). [file Image_4.tif]
