# Supplementary material for: Comparison of lobectomy and sublobar resection for stage I non-small cell lung cancer: a meta-analysis based on randomized controlled trials
Source: Front Oncol. 2023 Oct 4;13:1261263. doi: 10.3389/fonc.2023.1261263 (PMC10582352; doi:10.3389/fonc.2023.1261263)
Supplement: Supplementary file 5 [file Table_1.docx]

**Supplementary Table 1. Characteristics of all the studies included in the meta-analysis.**

| Author | Year | Trial registration number | Recruitment time | Mean Age  (years) | NO.  (Se: W) | Gender (Male/Female) | |
| --- | --- | --- | --- | --- | --- | --- | --- |
|  |  |  |  |  |  | Experiment | Control |
| Robert J. Ginsberg | 1995 | / | 1982.2-1988.11 | NA | 167/80 | NA | NA |
| Terumoto Koike | 2016 | / | 2005.2-2008.12 | NA | 30/2 | 11/22 | 20/12 |
| Nasser K Altorki | 2022 | NCT00499330 | 2007.6-2017.3 | 67.9 | 200/129 | 150/190 | 147/210 |
| Georgios Stamatis | 2022 | DRKS00004897 | 2013.10-2016.6 | 67 | 53/0 | 32/21 | 30/24 |
| Hisashi Saji | 2022 | UMIN000002317 | 2009.8-2014.10 | 67 | 552/0 | 290/262 | 293/261 |

NA, not available; W, wedge resection; Se, segmentectomy.
